# Roles of exosomal non-coding RNAs in osteoarthritis

**DOI:** 10.3389/fimmu.2026.1829319

**Published:** 2026-04-23

**Authors:** Jing Lu, Jiayou Wen, Jing Ji

**Affiliations:** 1 School of Traditional Chinese and Western Medicine, Gansu University of Chinese Medicine, Lanzhou, Gansu, China; 2 Department of Rehabilitation Medicine, Gansu Provincial Hospital of Traditional Chinese Medicine, Lanzhou, Gansu, China

**Keywords:** biomarkers, cartilage degradation, exosomes, inflammation, non-coding RNAs, osteoarthritis

## Abstract

Osteoarthritis (OA) is increasingly recognized as a chronic low-grade inflammatory joint disorder characterized by progressive cartilage degeneration, synovial inflammation, subchondral bone remodeling, and disrupted intercellular communication. Growing evidence indicates that exosomal non-coding RNAs (ncRNAs), including microRNAs, long non-coding RNAs, and circular RNAs, are key regulators of the OA microenvironment. By mediating intercellular signal transfer among chondrocytes, synovial fibroblasts, macrophages, osteoblasts, and osteoclasts, exosomal ncRNAs influence inflammatory mediator production, immune cell polarization, extracellular matrix metabolism, and osteochondral homeostasis. These regulatory effects are closely associated with major signaling pathways, including NF-κB, PI3K/AKT/mTOR, MAPK, and inflammasome-related cascades. Beyond their mechanistic roles in disease progression, exosomal ncRNAs also show strong potential as minimally invasive biomarkers for OA diagnosis and staging, as well as therapeutic agents or delivery vehicles for targeted intervention. This review focuses on the mechanisms of exosomal ncRNAs in modulating the osteoarthritis inflammation microenvironment, highlighting the potential of exosomal ncRNAs in osteoarthritis diagnosis and the prospects for their use in osteoarthritis medicine.

## 1 Introduction

OA is recognized as a chronic low-grade inflammatory disease involving persistent crosstalk among chondrocytes, synovial fibroblasts, macrophages, osteoblasts, osteoclasts, and extracellular matrix components (1, 2). This inflammatory milieu is characterized by aberrant production of cytokines and catabolic mediators, including interleukin-1β (IL-1β), tumor necrosis factor-α (TNF-α), interleukin-6 (IL-6), cyclooxygenase-2 (COX-2), prostaglandin E2 (PGE2), matrix metalloproteinases (particularly MMP-13), and a disintegrin and metalloproteinase with thrombospondin motifs (ADAMTS), which collectively drive cartilage matrix degradation, chondrocyte dysfunction, synovial inflammation, and subchondral bone remodeling (3, 4). Besides, these pathological processes are closely associated with activation of canonical inflammatory pathways, especially NF-κB, PI3K/AKT/mTOR, MAPK, and NLRP3 inflammasome signaling (5, 6).

Currently, ncRNAs have emerged as potential biomarkers or therapeutic targets for multiple diseases. Exosome-carried ncRNAs—mainly microRNAs (miRNAs), circular RNAs (circRNAs), and long non-coding RNAs (lncRNAs)—play a crucial role in modulating recipient cell activities and functions. They are significant in the pathogenesis of osteoarthritis and hold promise as biomarkers and therapeutic targets for diagnosis and treatment (7). Recent advances in exosome research and related technologies have expanded the potential for using exosomal ncRNAs in managing osteoarthritis. Existing literature indicates that exosomes and exosome-derived ncRNAs are widely present in osteoarthritis tissues and contribute to cartilage degeneration (8), though the specific mechanisms by which they influence disease progression via microenvironment-related pathways remain unclear. This review summarizes the role of exosomal ncRNAs in modulating the osteoarthritis microenvironment and their latest applications in clinical diagnosis and treatment, analyzing recent progress in understanding their mechanisms and targets to support future developments in osteoarthritis management.

## 2 Exosome-derived ncRNAs in osteoarthritis microenvironment: roles and mechanisms

### 2.1 Exosome-derived ncRNAs regulate inflammatory responses in osteoarthritis

#### 2.1.1 Exosome-derived ncRNAs regulate inflammatory factors

Exosome-derived ncRNAs regulate the production of inflammatory factors by influencing the expression of specific genes within cells. These ncRNAs can act as messenger RNAs, transmitted via exosomes to other cells, thereby affecting gene expression in target cells and regulating the synthesis and secretion of inflammatory factors (9, 10). Exosomes derived from adipose-derived mesenchymal stem cells transfer miR-5-338p, which targets Runt-related transcription factor 2, promoting chondrocyte proliferation and inhibiting the expression of prostaglandin E2, IL-6, IL-1β, and tumor necrosis factor-alpha (11). Moreover, exosome-derived miR-214-3p from synovial fibroblasts can reduce the expression of TNF-α, IL-1β, and interleukin-6 (12). These exosome-derived ncRNAs regulate the expression of inflammatory factors to suppress chondrocyte inflammatory damage and even maintain the morphology of subchondral bone.

In recipient chondrocytes and synovial cells, exosomal ncRNAs potentially modulate the activation state of the TLR4/MyD88/NF-κB axis, thereby altering the nuclear translocation of NF-κB subunits and the subsequent transcription of pro-inflammatory mediators such as IL-1β, IL-6, TNF-α, COX-2, and inducible nitric oxide synthase (13, 14). In parallel, exosomal ncRNAs may influence MAPK-dependent signaling, including the p38, JNK, and ERK branches, which are closely associated with inflammatory amplification, matrix-degrading enzyme induction, and cellular stress adaptation in osteoarthritic cartilage (15, 16). In addition, some exosomal ncRNAs are likely to participate in the post-transcriptional fine-tuning of JAK/STAT signaling, thereby affecting the responsiveness of joint-resident cells to inflammatory cytokines and reinforcing chronic low-grade synovitis (17, 18). Exosomal ncRNAs may also regulate redox-sensitive pathways and mitochondrial stress responses, which are increasingly recognized as critical drivers of inflammatory factor release and matrix catabolism in OA (19, 20).

Moreover, exosome-derived ncRNAs activate inflammasomes during the progression of osteoarthritis. Inflammasomes are intracellular multimeric structures that regulate the inflammatory response within cells. Exosome-derived ncRNAs can intervene in inflammasome activation by modulating the expression of genes or proteins involved in related pathways (21). Studies have shown that exosome-derived miR-449a-5p from chondrocytes in osteoarthritis patients can induce inflammasome activation in cell lines and accelerate cartilage erosion in osteoarthritis mouse models (22). The activation of inflammasomes is a critical step in the process of pyroptosis, which, in turn, releases inflammatory factors, exacerbating intra-articular inflammation and forming a vicious cycle (23, 24). Due to the critical roles of inflammatory factors and inflammasomes in the pathogenesis of osteoarthritis, ncRNAs regulating these upstream factors are likely involved in initiating chondrocyte death and matrix degeneration in the osteoarthritis microenvironment (25, 26).

#### 2.1.2 Crosstalk between exosome-derived ncRNAs and immune cell in osteoarthritis

**Figure 1:**
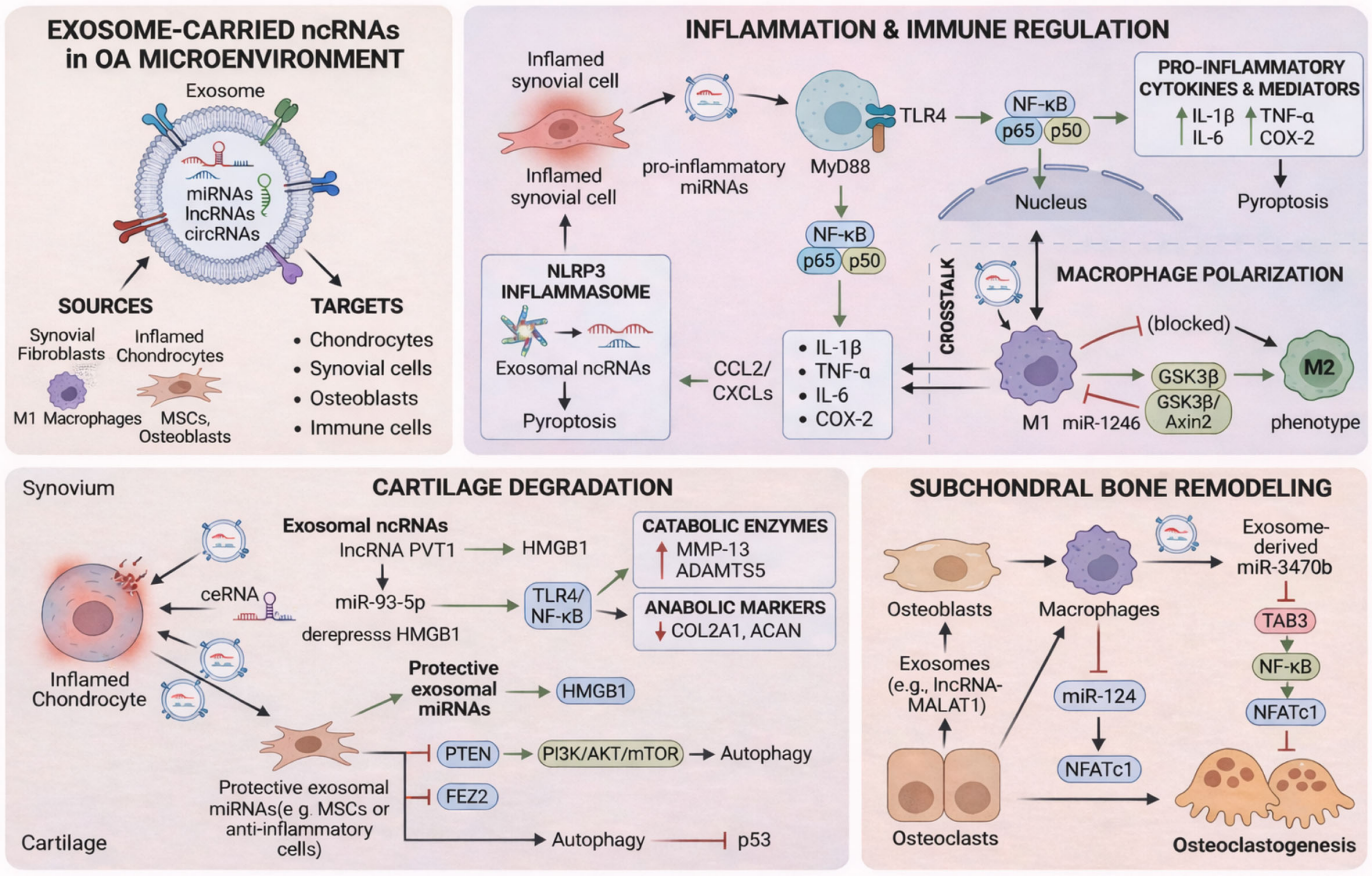
Roles of exosomal non-coding RNAs in the pathogenesis, diagnosis, and treatment of osteoarthritis. Exosomal non-coding RNAs (ncRNAs), including miRNAs, lncRNAs, and circRNAs, are secreted by synovial fibroblasts, inflamed chondrocytes, macrophages, mesenchymal stem cells, osteoblasts, and osteoclasts, and transferred to recipient cells within the joint microenvironment. These exosomal cargos regulate inflammatory signaling, macrophage polarization, inflammasome activation, pyroptosis, cartilage matrix metabolism, autophagy, and subchondral bone remodeling through pathways such as TLR4/MyD88/NF-κB, HMGB1/TLR4/NF-κB, PI3K/AKT/mTOR, and NFATc1-related signaling. In cartilage, exosomal ncRNAs modulate catabolic enzymes and anabolic markers, thereby influencing extracellular matrix degradation and chondrocyte homeostasis. In subchondral bone, they participate in osteoblast–macrophage–osteoclast crosstalk and osteoclastogenesis.

Exosomal ncRNAs regulate inflammatory responses by affecting the activity of immune cells. For example, they can play a role in modulating the functions of immune cells such as macrophages, thereby influencing the intensity and nature of immune responses. Studies have shown that downregulation of M1 macrophage-derived exosomal miR-146b-5p can target Usp3 and Sox5, alleviating the inflammatory microenvironment of osteoarthritis and promoting cartilage formation both *in vitro* and *in vivo* (27). Exosomal miR-6 derived from human umbilical mesenchymal stem cells interacts with METTL3 to reduce m6A modifications on macrophage NLRP3, thereby inhibiting the secretion of pro-inflammatory factors and the degradation of extracellular matrix in cartilage cells, which alleviates osteoarthritis in murine knee joints (28). Similarly, exosomal miR-486-5p can regulate macrophage polarization (29) to alleviate chondrocyte apoptosis. In studies investigating how exosomal ncRNAs affect the intra-articular environment via other immune cells, such as lymphocytes and neutrophils, it has been observed that exosomal ncRNAs can alleviate the inflammatory responses they participate in (30), although the specific mechanisms remain unclear. Exosome-derived ncRNAs regulate macrophage efferocytosis and apoptotic cell clearance, thereby influencing the persistence of danger-associated molecular patterns in synovial tissues (17, 31, 32). ncRNA cargos may modulate the MerTK-dependent phagocytic program and its downstream PI3K-AKT-STAT6 signaling cascade, promoting a pro-resolving macrophage phenotype and limiting secondary inflammatory amplification (33, 34). Nevertheless, exosomal ncRNAs may influence the CD39/CD73–adenosine axis, which converts extracellular ATP into immunosuppressive adenosine and subsequently activates A2A receptor signaling in immune cells, thereby dampening excessive synovial immune activation and restraining tissue-destructive inflammation (35, 36). Importantly, exosome-derived ncRNAs may reshape immune-cell recruitment and retention by modulating chemokine networks such as the CCL2/CCR2 axis and CXCL-mediated leukocyte trafficking, thereby limiting the accumulation of inflammatory monocytes and neutrophils in the synovium (37, 38). Therefore, as research into immune regulation in disease progression deepens, exosomal ncRNAs may target immune system modulation to regulate inflammatory responses in osteoarthritis. Future studies could further explore how exosomal ncRNAs are associated with the immune activation of different immune cells (**Figure 1**).

#### 2.1.3 Exosome-derived ncRNAs suppress the inflammatory response in osteoarthritis

Exosomal ncRNAs modulate multiple intracellular signaling cascades, thereby shaping chondrocyte responses to inflammatory stimuli through either activation or suppression of specific pathways. For example, exosomes derived from hypoxia-preconditioned bone marrow mesenchymal stem cells promote chondrocyte proliferation, migration and anabolic metabolism, while suppressing intra-articular inflammation through the miR-205-5p/PTEN/AKT axis (39). Beyond mediating stromal–chondrocyte crosstalk, exosomes secreted by chondrocytes themselves also participate in the pathological progression of osteoarthritis and in local intercellular communication. Notably, exosomal lncRNAs released from injured chondrocytes have been shown to activate the PI3K/AKT/mTOR pathway, thereby modulating intra-articular inflammation and exacerbating joint degeneration in rat models (40). As the signaling architecture underlying joint degeneration becomes increasingly defined, exosomal ncRNAs are emerging as upstream regulatory nodes that connect inflammatory and degenerative pathways. Thus, they may constitute a pivotal link within the broader “exosome–signalling pathway–inflammatory response/chondrocyte programmed or non-programmed cell death” axis, although the relevant mechanisms remain incompletely understood (41). Given that inflammation is central to osteoarthritis progression, the ability of exosomal ncRNAs to regulate intra-articular inflammation through diverse targets and mechanisms further supports their potential as therapeutic entry points for suppressing disease-associated inflammatory responses (42).

### 2.2 Exosomal ncRNAs regulate cartilage degradation

Articular cartilage is primarily composed of chondrocytes and the extracellular matrix. Chondrocytes, as the unique cells in cartilage tissue, regulate the synthesis and metabolism of the extracellular matrix. Firstly, ncRNAs in exosomes can influence the function and survival of chondrocytes, thereby slowing down cartilage degeneration. In the inhibition of chondrocyte senescence, ncRNAs can restore senescent chondrocytes by regulating p53-related pathways. For example, miRNAs (miR-let-7i-5p) in exosomes from umbilical mesenchymal stem cells target the p53 signaling pathway in chondrocytes, promoting cartilage repair (43). Additionally, miR-429 in exosomes from adipose stem cells promotes chondrocyte autophagy by targeting FEZ2, thereby improving cartilage damage (44). Beyond autophagy, apoptosis, pyroptosis, ferroptosis, and other cell death pathways in chondrocytes are closely linked to exosomal ncRNAs.

Exosomal ncRNAs derived from synovial fibroblasts or inflamed chondrocytes can activate or suppress key inflammatory cascades that converge on cartilage destruction, including the TLR4/TRAF6/NF-κB, HMGB1/TLR4/NF-κB, and Trim14/NF-κB/IFN-β axes (45, 46). These pathways ultimately regulate the expression of catabolic mediators such as MMP13 and ADAMTS5, while suppressing anabolic markers including COL2A1 and ACAN, thereby accelerating extracellular matrix breakdown (20, 47, 48). Studies summarized in recent OA exosome literature show that exosomal lncRNA PVT1 can function as a competing endogenous RNA for miR-93-5p, thereby derepressing HMGB1 and activating TLR4/NF-κB signaling, which promotes inflammatory injury and collagen degradation in chondrocytes (49, 50). Likewise, exosomes derived from fibroblast-like synoviocytes enriched in miR-146a attenuate cartilage degeneration by targeting TRAF6 and suppressing the TLR4/TRAF6/NF-κB pathway, while simultaneously shifting synovial macrophages away from the pro-inflammatory M1 phenotype (20, 51). In parallel, normal fibroblast-like synoviocyte-derived exosomal miR-150-3p has been reported to inhibit the Trim14/NF-κB/IFN-β innate immune axis, thereby reducing chondrocyte inflammatory activation and delaying OA progression (20, 52). These findings suggest that exosomal ncRNAs can influence cartilage degradation not only by regulating chondrocyte viability, but also by rewiring innate immune signaling at the synovium–cartilage interface.

In addition, immune cell-derived exosomal ncRNAs further amplify the inflammatory-catabolic coupling that underlies cartilage loss. For example, M1 macrophage-derived exosomal miR-1246 has been shown to activate the Wnt/β-catenin pathway in chondrocytes through suppression of GSK3β and Axin2, thereby promoting inflammatory responses and matrix injury (53, 54). By contrast, protective exosomal cargos from anti-inflammatory cellular sources can restrain these destructive programs and preserve cartilage homeostasis (55). Exosomal ncRNAs also modulate chondrocyte apoptosis and degeneration by reshaping gene expression and extracellular matrix interactions. For example, exosomal miR-3-122p derived from hypoxic adipose stem cells enhances cartilage matrix synthesis, including proteoglycans and type II collagen, in osteoarthritis-like inflammatory chondrocytes, while suppressing inflammatory mediators (CEBPβ, cyclooxygenase-2, interleukin-6 and tumor necrosis factor-α) and catabolic factors such as matrix metalloproteinase-13 and ADAMTS5 (56). Regulation of extracellular matrix homeostasis by exosomal ncRNAs is likewise crucial for preserving chondrocyte integrity. Decellularized extracellular matrix can provide an optimal niche for mesenchymal stem cell expansion (57). Within this microenvironment, miR-3473b-enriched exosomes target PTEN to activate PTEN/AKT signaling, promoting cartilage matrix migration, enhancing anabolic metabolism, and inhibiting chondrocyte apoptosis, ultimately alleviating osteoarthritis (58, 59).

### 2.3 Exosomal ncRNAs regulate subchondral bone remodeling

Subchondral bone is one of the fundamental units that constitutes joint structure and function. As the cartilage matrix wears down, the pressure exerted on the subchondral bone increases, disrupting the balance between osteoclasts and osteoblasts (60), leading to the absorption and remodeling of the subchondral bone. Exosomal ncRNAs can interact with bone cells, regulating the differentiation and function of osteoblasts in the bone marrow stroma, thereby influencing the process of bone resorption and remodeling. Studies have shown that exosomal lncRNA-MALAT1, derived from osteoblasts, promotes osteoclastogenesis by targeting the miR-124/NFATc1 signaling axis in bone marrow-derived macrophages (61). Osteoblasts and mature osteoclasts capture macrophage-derived exosomes, with exosomal miR-3470b targeting the TAB3/NF-κB signaling pathway to increase NFATc1 expression and induce osteoclast differentiation (62). Moreover, they also demonstrated that exosomes enriched with miR-3470b help inhibit bone resorption. Meanwhile, exosomal ncRNAs derived from subchondral bone regulate chondrocytes and the cartilage matrix, with their transport routes potentially reaching cartilage through microfractures or blood vessels, reflecting the intercellular communication role of exosomal ncRNAs within joint cells (63).

### 2.4 Exosomal ncRNAs regulate intercellular communication

Exosomal ncRNAs function as potent intercellular messengers, transmitting molecular signals that regulate the behavior of cells both adjacent to and distant from the lesion site. Such communication is increasingly recognized as a key driver of osteoarthritis pathogenesis. Liu et al. (64) showed that mechanical stretch stimulates chondrocytes to secrete exosomal miR-9-5p, which suppresses osteoblast differentiation by targeting KLF5 (65). Through the circStrn3/miR-9-5p/KLF5 axis, this regulatory network also restrains matrix metabolism in chondrocytes, thereby delaying osteoarthritis progression. In parallel, cells from the infrapatellar fat pad release abundant exosomes enriched in miR-100-5p (66), which preserve cartilage homeostasis by inhibiting the mTOR-dependent autophagy pathway. Importantly, the regulatory scope of exosomal ncRNAs is not confined to joint-resident or periarticular tissues. Exosomal ncRNAs derived from distant tissues can likewise engage in molecular crosstalk with joint cells (67). For example, exosomal miR-140-5p from human urine-derived stem cells alleviates knee osteoarthritis in rats by downregulating vascular endothelial growth factor A, whereas exosomes from human umbilical mesenchymal stem cells suppress reactive oxygen species production and chondrocyte apoptosis in human articular chondrocytes through the miR-100-5p/NOX4 axis (68). Collectively, these findings underscore the central role of exosomal ncRNAs in mediating local and systemic intercellular communication, possibly through their entry into the circulation and subsequent participation in systemic metabolic regulation (69). This further indicates their substantial translational potential as diagnostic biomarkers and therapeutic targets in osteoarthritis and even broader systemic disorders (70).

## 3 Exosomal ncRNAs in osteoarthritis diagnosis and treatment

### 3.1 Exosomal ncRNAs as biomarkers of osteoarthritis diagnosis

Exosome-derived ncRNAs have emerged as one of the most promising cell-free strategies for the management of bone- and joint-related musculoskeletal disorders (71). Their translational appeal is largely attributable to their high circulatory stability, favorable biocompatibility, and low immunogenicity and toxicity (72). At present, however, the diagnosis of osteoarthritis (OA) still relies predominantly on imaging modalities such as MRI, while robust molecular biomarkers for definitive diagnosis remain lacking (73). Exosomal ncRNAs can serve as biomarkers for osteoarthritis, aiding in early diagnosis and monitoring disease progression and prognosis. Their expression levels may correlate with the severity of the disease and inflammation (74). RUNX2-derived hsa_circ_0005526 (circ_RUNX2) was significantly overexpressed in the serum of OA patients (75). Circ_RUNX2 may participate in the development of OA by interacting with cartilage matrix receptors, making it a potential clinical indicator for osteoarthritis (76). In addition, ncRNAs in synovial fluid may serve as indicators of intra-articular injury and disease stage. For example, exosomal lncRNA PCGEM1 is highly expressed in patients with OA and exhibits stage-dependent differential expression (77). Subsequent studies further suggest that characteristic OA-associated ncRNAs may be linked to histopathological alterations and disease grade (78).

### 3.2 Exosomal ncRNAs in osteoarthritis treatment

As central regulators of osteoarthritis pathobiology, exosomal ncRNAs participate in multiple molecular pathways implicated in disease initiation and progression, thereby emerging as attractive therapeutic targets (79). Currently, several studies are exploring the use of RNA or drugs to intervene in the expression of these ncRNAs to alleviate arthritis inflammation and pain (80). Additionally, exosomes can serve as potential drug delivery vehicles to transport therapeutic ncRNAs or drugs to the osteoarthritis lesions, thereby improving treatment efficacy and reducing adverse reactions (81–84). For instance, exosomal miRNA-100-5p inhibits intra-articular inflammation via the miR-100-5p/mTOR pathway (85); exosomal miR-92a-3p induces the expression of cartilage markers (type II collagen and proteoglycans) while inhibiting the degradation of matrix metalloproteinase-13 (86); and exosomal miRNA-29A-3p and miRNA-93-5P target subchondral bone and immune cells to alleviate intra-articular inflammatory responses (87). Therefore, these findings suggest that future studies should prioritize strategies that enhance the abundance of endogenous exosomal ncRNAs with anti-inflammatory or tissue-protective functions, while limiting the release of exosomal ncRNAs that drive inflammation or participate in catabolic pathway.

### 3.3 Exosomal ncRNA delivery strategy for osteoarthritis

Exosomes have attracted sustained interest as drug delivery platforms because they can penetrate the dense, avascular extracellular matrix of cartilage with high efficiency (88). Accordingly, increasing efforts have focused on exploiting exosomes to deliver therapeutic ncRNAs and thereby attenuate osteoarthritis progression, particularly through intra-articular miRNA delivery. One representative strategy involved fusing a cartilage-affinity peptide (CAP) to exosomal lysosome-associated membrane glycoprotein 2b on the exosomal surface, generating CAP-engineered exosomes capable of efficiently loading miR-140 (89). These exosomes delivered miR-140 into deep cartilage regions across the dense middle zone, suppressed cartilage-degrading proteases and alleviated osteoarthritis progression in rat models. More recently, mesenchymal stem cell-derived exosomes isolated from human subcutaneous adipose tissue obtained by liposuction have been engineered to enable targeted delivery of miR-199a-3p into chondrocytes, with favorable therapeutic efficacy *in vivo* (90). Meanwhile, advances in tissue engineering have further expanded the therapeutic potential of exosomal ncRNAs. Notably, the combination of exosomes carrying circRNA3503 with an injectable thermosensitive hydrogel has been proposed as an effective targeted strategy for osteoarthritis prevention (91). Nevertheless, methods for intra-articular exosome delivery remain under active investigation, and the development of more stable and efficient scaffolds or biomaterials will be essential for optimizing exosome-based therapy (92).

## 4 Conclusion

In conclusion, exosomal non-coding RNAs have emerged as central regulators of the osteoarthritic microenvironment by orchestrating inflammatory signaling, cartilage homeostasis, subchondral bone remodeling, and intercellular communication. Rather than acting as passive molecular byproducts, these cargos function as active signaling mediators that influence the behavior of chondrocytes, synovial fibroblasts, macrophages, osteoblasts, and osteoclasts. Through modulation of pathways such as NF-κB, PI3K/AKT/mTOR, MAPK, and inflammasome-associated signaling, exosomal ncRNAs can either exacerbate or alleviate chronic low-grade inflammation, matrix degradation, and progressive joint destruction. Their dual role in immune regulation and tissue remodeling underscores the mechanistic importance of exosome-mediated molecular exchange in OA pathogenesis.

From a translational perspective, exosomal ncRNAs hold substantial promise as minimally invasive biomarkers and as next-generation therapeutic tools for precision management of osteoarthritis. Their relative stability in biological fluids, low immunogenicity, and intrinsic capacity for targeted delivery make them attractive candidates for disease monitoring and intervention. Nevertheless, major challenges remain, including cargo heterogeneity, incomplete mechanistic mapping, limitations in large-scale isolation and standardization, and the need to improve targeting efficiency and long-term safety. Future studies should integrate multi-omics profiling, bioengineering strategies, and clinically relevant models to define functional ncRNA networks and accelerate the development of exosome-based diagnostic and therapeutic platforms for OA.

## References

[1] De Roover A Escribano-Núñez A Monteagudo S Lories R . Fundamentals of osteoarthritis: Inflammatory mediators in osteoarthritis. Osteoarthritis Cartilage. (2023) 31:1303–11. doi: 10.1016/j.joca.2023.06.005. PMID: 37353140

[2] Chen B Sun Y Xu G Jiang J Zhang W Wu C . Role of crosstalk between synovial cells and chondrocytes in osteoarthritis (review). Exp Ther Med. (2024) 27:201. doi: 10.3892/etm.2024.12490. PMID: 38590580 PMC11000048

[3] Mukherjee A Das B . The role of inflammatory mediators and matrix metalloproteinases (MMPs) in the progression of osteoarthritis. Biomater Biosyst. (2024) 13:100090. doi: 10.1016/j.bbiosy.2024.100090. PMID: 38440290 PMC10910010

[4] Hu Y Chen X Wang S Jing Y Su J . Subchondral bone microenvironment in osteoarthritis and pain. Bone Res. (2021) 9:20. doi: 10.1038/s41413-021-00147-z. PMID: 33731688 PMC7969608

[5] Batarfi WA Yunus MHM Hamid AA Maarof M Abdul Rani R . Breaking down osteoarthritis: Exploring inflammatory and mechanical signaling pathways. Life (Basel). (2025) 15:1238. doi: 10.3390/life15081238. PMID: 40868886 PMC12387175

[6] Liu Y Wang Y Yan P Cui N Xu K Liu D . NLRP3 inflammasome-mediated osteoarthritis: The role of epigenetics. (2025) 14:71. doi: 10.3390/biology14010071 PMC11761621 39857301

[7] Ali SA Peffers MJ Ormseth MJ Jurisica I Kapoor M . The non-coding RNA interactome in joint health and disease. Nat Rev Rheumatol. (2021) 17:692–705. doi: 10.1038/s41584-021-00687-y. PMID: 34588660

[8] Tu C He J Chen R Li Z . The emerging role of exosomal non-coding RNAs in musculoskeletal diseases. Curr Pharm Des. (2019) 25:4523–35. doi: 10.2174/1381612825666191113104946. PMID: 31724510

[9] Lai Z Ye T Zhang M Mu Y . Exosomes as vehicles for noncoding RNA in modulating inflammation: A promising regulatory approach for ischemic stroke and myocardial infarction. J Inflammation Res. (2024) 17:7485–501. doi: 10.2147/jir.s484119. PMID: 39464334 PMC11505480

[10] Lai X Zhong J Zhang B Zhu T Liao R . Exosomal non-coding RNAs: Novel regulators of macrophage-linked intercellular communication in lung cancer and inflammatory lung diseases. Biomolecules. (2023) 13:536. doi: 10.3390/biom13030536. PMID: 36979471 PMC10046066

[11] Li C Li W Pu G Wu J Qin F . Exosomes derived from miR-338-3p-modified adipose stem cells inhibited inflammation injury of chondrocytes via targeting RUNX2 in osteoarthritis. J Orthop Surg Res. (2022) 17:567. doi: 10.1186/s13018-022-03437-2. PMID: 36572886 PMC9791748

[12] Lai C Liao B Peng S Fang P Bao N Zhang L . Synovial fibroblast-miR-214-3p-derived exosomes inhibit inflammation and degeneration of cartilage tissues of osteoarthritis rats. Mol Cell Biochem. (2023) 478:637–49. doi: 10.1007/s11010-022-04535-9. PMID: 36001206 PMC9938056

[13] Xue HY Shen XL Wang ZH Bi HC Xu HG Wu J . Research progress on mesenchymal stem cell-derived exosomes in the treatment of osteoporosis induced by knee osteoarthritis (review). Int J Mol Med. (2025) 56:160. doi: 10.3892/ijmm.2025.5601. PMID: 40747674 PMC12339181

[14] Chen P Zhou J Ruan A Guan H Xie J Zeng L . Synovial tissue-derived extracellular vesicles induce chondrocyte inflammation and degradation via NF-κB signaling pathway: An *in vitro* study. J Cell Mol Med. (2022) 26:2038–48. doi: 10.1111/jcmm.17227. PMID: 35179308 PMC8980928

[15] Lan S Zhang C . Roles of exosomes in immune regulation of osteoarthritis and their applications in inflammation repair. (2025) 16:1611718. doi: 10.3389/fimmu.2025.1611718 PMC12443577 40977699

[16] Li Z Dai A Yang M Chen S Deng Z Li L . p38MAPK signaling pathway in osteoarthritis: Pathological and therapeutic aspects. J Inflammation Res. (2022) 15:723–34. doi: 10.2147/jir.s348491. PMID: 35140502 PMC8820459

[17] Sadeghi M Tavakol Afshari J Fadaee A Dashti M Kheradmand F Dehnavi S . Exosomal miRNAs involvement in pathogenesis, diagnosis, and treatment of rheumatoid arthritis. Heliyon. (2025) 11:e41983. doi: 10.1016/j.heliyon.2025.e41983. PMID: 39897907 PMC11786886

[18] Wu L-F Zhang Q Mo X-B Lin J Wu Y-L Lu X . Identification of novel rheumatoid arthritis-associated MiRNA-204-5p from plasma exosomes. Exp Mol Med. (2022) 54:334–45. doi: 10.1038/s12276-022-00751-x. PMID: 35354913 PMC8980013

[19] Chen J Yu X Zhang X . Advances on biological functions of exosomal non-coding RNAs in osteoarthritis. Cell Biochem Funct. (2022) 40:49–59. doi: 10.1002/cbf.3679. PMID: 34921424

[20] Chen P Zeng L Wang T He J Xiong S Chen G . The communication role of extracellular vesicles in the osteoarthritis microenvironment. Front Immunol. (2025) 16:1549833. doi: 10.3389/fimmu.2025.1549833. PMID: 40165965 PMC11955493

[21] Hegde M Kumar A Girisa S Alqahtani MS Abbas M Goel A . Exosomal noncoding RNA-mediated spatiotemporal regulation of lipid metabolism: Implications in immune evasion and chronic inflammation. Cytokine Growth Factor Rev. (2023) 73:114–34. doi: 10.1016/j.cytogfr.2023.06.001. PMID: 37419767

[22] Noonin C Thongboonkerd V . Exosome-inflammasome crosstalk and their roles in inflammatory responses. Theranostics. (2021) 11:4436–51. doi: 10.7150/thno.54004. PMID: 33754070 PMC7977448

[23] Du X Liu R Jiang Z Zhang C Yang Z Hu S . Chondrocyte lysates activate NLRP3 inflammasome-induced pyroptosis in synovial fibroblasts to exacerbate knee synovitis by downregulating caveolin-1. Arthritis Res Ther. (2025) 27:104. doi: 10.1186/s13075-025-03573-0. PMID: 40375346 PMC12083164

[24] Yang F Li D Long W Li E Wei B . Role of pyroptosis in the pathogenesis of osteoarthritis: An updated review. J Inflammation Res. (2025) 18:15065–79. doi: 10.2147/jir.s547458. PMID: 41185687 PMC12579881

[25] Chen X Jiang Q Ren L Ren H Xu H Wang J . BET proteins inhibitor JQ1 impairs GM-CSF-promoted peritoneal macrophage self-renewal and IL-4-induced alternative polarization. Int Immunopharmacol. (2023) 124:110942. doi: 10.1016/j.intimp.2023.110942. PMID: 37716160

[26] Tang S Cao Y Cai Z Nie X Ruan J Zhou Z . The lncRNA PILA promotes NF-κB signaling in osteoarthritis by stimulating the activity of the protein arginine methyltransferase PRMT1. Sci Signal. (2022) 15:eabm6265. doi: 10.1126/scisignal.abm6265. PMID: 35609127

[27] Jia H Duan L Yu P Zhou Y Liu R Wang H . Digoxin ameliorates joint inflammatory microenvironment by downregulating synovial macrophage M1-like-polarization and its-derived exosomal miR-146b-5p/Usp3&Sox5 axis. Int Immunopharmacol. (2022) 111:109135. doi: 10.1016/j.intimp.2022.109135. PMID: 35987145

[28] Zhou H Shen X Yan C Xiong W Ma Z Tan Z . Extracellular vesicles derived from human umbilical cord mesenchymal stem cells alleviate osteoarthritis of the knee in mice model by interacting with METTL3 to reduce m6A of NLRP3 in macrophage. Stem Cell Res Ther. (2022) 13:322. doi: 10.1186/s13287-022-03005-9. PMID: 35842714 PMC9288728

[29] Wang Y Fan A Lu L Pan Z Ma M Luo S . Exosome modification to better alleviates endoplasmic reticulum stress induced chondrocyte apoptosis and osteoarthritis. Biochem Pharmacol. (2022) 206:115343. doi: 10.1016/j.bcp.2022.115343. PMID: 36370754

[30] Huang L Dong G Peng J Li T Zou M Hu K . The role of exosomes and their enhancement strategies in the treatment of osteoarthritis. Hum Cell. (2023) 36:1887–900. doi: 10.1007/s13577-023-00970-y. PMID: 37603220

[31] Toussirot E Bonnefoy F Vauchy C Perruche S Saas P . Mini-review: The administration of apoptotic cells for treating rheumatoid arthritis: Current knowledge and clinical perspectives. (2021) 12:630170. doi: 10.3389/fimmu.2021.630170 PMC7950318 33717160

[32] Dong X Peng S Ling Y Huang B Tu W Sun X . ATRA treatment slowed P-selectin-mediated rolling of flowing HL60 cells in a mechano-chemical-dependent manner. Front Immunol. (2023) 14:1148543. doi: 10.3389/fimmu.2023.1148543. PMID: 37168856 PMC10164934

[33] Tie YF Liu H Zhang T Meng TW Liang Q . Dual role and clinical application of extracellular vesicles in acute respiratory distress syndrome: Mechanism analysis and translational challenges. World J Stem Cells. (2025) 17:108657. doi: 10.4252/wjsc.v17.i9.108657. PMID: 41025111 PMC12476792

[34] Chen F Li Y Zhao L Lin C Zhou Y Ye W . Anti-inflammatory effects of MerTK by inducing M2 macrophage polarization via PI3K/Akt/GSK-3β pathway in gout. Int Immunopharmacol. (2024) 142:112942. doi: 10.1016/j.intimp.2024.112942. PMID: 39217874

[35] Lu JC Zhang PF Huang XY Guo XJ Gao C Zeng HY . Amplification of spatially isolated adenosine pathway by tumor-macrophage interaction induces anti-PD1 resistance in hepatocellular carcinoma. J Hematol Oncol. (2021) 14:200. doi: 10.1186/s13045-021-01207-x. PMID: 34838121 PMC8627086

[36] Ortiz MA Diaz-Torné C De Agustin JJ Estrada P Reina D Hernandez MV . Altered CD39 and CD73 expression in rheumatoid arthritis: Implications for disease activity and treatment response. Biomolecules. (2023) 14:1. doi: 10.3390/biom14010001. PMID: 38275742 PMC10813161

[37] Saadh MJ Allela OQB Al-Hussainy AF Baldaniya L Rekha MM Nathiya D . Exosomal non-coding RNAs: Gatekeepers of inflammation in autoimmune disease. J Inflammation (Lond). (2025) 22:18. doi: 10.1186/s12950-025-00443-z. PMID: 40369549 PMC12079953

[38] Wright HL Lyon M Chapman EA Moots RJ Edwards SW . Rheumatoid arthritis synovial fluid neutrophils drive inflammation through production of chemokines, reactive oxygen species, and neutrophil extracellular traps. (2021) 11:584116. doi: 10.3389/fimmu.2020.584116 PMC7813679 33469455

[39] Shen K Duan A Cheng J Yuan T Zhou J Song H . Exosomes derived from hypoxia preconditioned mesenchymal stem cells laden in a silk hydrogel promote cartilage regeneration via the miR-205-5p/PTEN/AKT pathway. Acta Biomater. (2022) 143:173–88. doi: 10.1016/j.actbio.2022.02.026. PMID: 35202856

[40] Lv G Wang B Li L Li Y Li X He H . Exosomes from dysfunctional chondrocytes affect osteoarthritis in Sprague-Dawley rats through FTO-dependent regulation of PIK3R5 mRNA stability. Bone Joint Res. (2022) 11:652–68. doi: 10.1302/2046-3758.119.bjr-2021-0443.r2. PMID: 36066338 PMC9533253

[41] Jiang S Tian G Yang Z Gao X Wang F Li J . Enhancement of acellular cartilage matrix scaffold by Wharton’s jelly mesenchymal stem cell-derived exosomes to promote osteochondral regeneration. Bioact Mater. (2021) 6:2711–28. doi: 10.1016/j.bioactmat.2021.01.031. PMID: 33665503 PMC7895679

[42] Liu Y Zeng Y Si HB Tang L Xie HQ Shen B . Exosomes derived from human urine-derived stem cells overexpressing miR-140-5p alleviate knee osteoarthritis through downregulation of VEGFA in a rat model. Am J Sports Med. (2022) 50:1088–105. doi: 10.1177/03635465221073991. PMID: 35179989

[43] Cao H Chen M Cui X Liu Y Liu Y Deng S . Cell-free osteoarthritis treatment with sustained-release of chondrocyte-targeting exosomes from umbilical cord-derived mesenchymal stem cells to rejuvenate aging chondrocytes. ACS Nano. (2023) 17:13358–76. doi: 10.1021/acsnano.3c01612. PMID: 37439514

[44] Meng C Na Y Han C Ren Y Liu M Ma P . Exosomal miR-429 derived from adipose-derived stem cells ameliorated chondral injury in osteoarthritis via autophagy by targeting FEZ2. Int Immunopharmacol. (2023) 120:110315. doi: 10.1016/j.intimp.2023.110315. PMID: 37245297

[45] González-Rodríguez A De Toro FJ Jorge-Mora A Fernandez-Pernas P Rivadulla CP Fraga M . Targeting osteoarthritis with small extracellular vesicle therapy: Potential and perspectives. Front Bioeng Biotechnol. (2025) 13:1570526. doi: 10.3389/fbioe.2025.1570526 40621210 PMC12226547

[46] Yang YI Wang YY Ahn JH Kim BH Choi JH . CCL2 overexpression is associated with paclitaxel resistance in ovarian cancer cells via autocrine signaling and macrophage recruitment. BioMed Pharmacother. (2022) 153:113474. doi: 10.1016/j.biopha.2022.113474. PMID: 36076499

[47] Li W Tao C Mao M Zhu K . The Nrf2/HMGB1/NF-κB axis modulates chondrocyte apoptosis and extracellular matrix degradation in osteoarthritis. Acta Biochim Biophys Sin (Shanghai). (2023) 55:818–30. doi: 10.3724/abbs.2023078. PMID: 37232576 PMC10281874

[48] Wang H Shu J Zhang C Wang Y Shi R Yang F . Extracellular vesicle-mediated miR-150-3p delivery in joint homeostasis: A potential treatment for osteoarthritis? Cells. (2022) 11:2766. doi: 10.3390/cells11172766. PMID: 36078172 PMC9454967

[49] Meng Y Qiu S Sun L Zuo J . Knockdown of exosome-mediated lnc-PVT1 alleviates lipopolysaccharide-induced osteoarthritis progression by mediating the HMGB1/TLR4/NF-κB pathway via miR-93-5p. Mol Med Rep. (2020) 22:5313–25. doi: 10.3892/mmr.2020.11594 PMC7646997 33174011

[50] Miao C Zhou W Wang X Fang J . The research progress of exosomes in osteoarthritis, with particular emphasis on the mediating roles of miRNAs and lncRNAs. (2021) 12:685623. doi: 10.3389/fphar.2021.685623 PMC8176107 34093208

[51] Wang H Zhang Y Zhang C Zhao Y Shu J Tang X . Exosomes derived from miR-146a-overexpressing fibroblast-like synoviocytes in cartilage degradation and macrophage M1 polarization: a novel protective agent for osteoarthritis? (2024) 15:1361606. doi: 10.3389/fimmu.2024.1361606 PMC11153682 38846937

[52] Jiang Y Xiao F Wang L Wang T Chen L . Correction to: Circular RNA hsa_circ_0000034 accelerates retinoblastoma advancement through the miR-361-3p/ADAM19 axis. Mol Cell Biochem. (2022) 477:1321. doi: 10.1007/s11010-021-04311-1. PMID: 35061160

[53] Peng S Yan Y Li R Dai H Xu J . Extracellular vesicles from M1-polarized macrophages promote inflammation in the temporomandibular joint via miR-1246 activation of the Wnt/β-catenin pathway. Ann N Y Acad Sci. (2021) 1503:48–59. doi: 10.1111/nyas.14590. PMID: 33759195

[54] Cui RM Zheng M Hong JB Wang ZX Cun YF Gao SJ . Research progress on the effects of macrophage-derived exosomes on muscle factors IGF-1 and FGF-2 mediating musculoskeletal crosstalk molecular signaling pathway on bone metabolism (Review). Int J Mol Med. (2026) 57:67. doi: 10.3892/ijmm.2026.5738. PMID: 41574692 PMC12851855

[55] Li Y Fu T Yu W Wen H Wang Z Lyu Z . Mesenchymal stem cells and extracellular vesicles for knee osteoarthritis: clinical application, mechanism exploration and prospect. Stem Cell Res Ther. (2025) 16:688. doi: 10.1186/s13287-025-04783-8. PMID: 41276868 PMC12751560

[56] Chang LH Wu SC Chen CH Chen JW Huang WC Wu CW . Exosomes derived from hypoxia-cultured human adipose stem cells alleviate articular chondrocyte inflammaging and post-traumatic osteoarthritis progression. Int J Mol Sci. (2023) 24:13414. doi: 10.3390/ijms241713414. PMID: 37686220 PMC10487932

[57] Wu X Lu W Xu C Jiang C Zhuo Z Wang R . Macrophages phenotype regulated by IL-6 are associated with the prognosis of platinum-resistant serous ovarian cancer: integrated analysis of clinical trial and omics. J Immunol Res. (2023) 2023:6455704. doi: 10.1155/2023/6455704. PMID: 37124547 PMC10132904

[58] Zhang Y Qi G Yan Y Wang C Wang Z Jiang C . Exosomes derived from bone marrow mesenchymal stem cells pretreated with decellularized extracellular matrix enhance the alleviation of osteoarthritis through miR-3473b/phosphatase and tensin homolog axis. J Gene Med. (2023) 25:e3510. doi: 10.1002/jgm.3510. PMID: 36998238

[59] Yang XH Chen SY Zhou QF Cai YZ . Exosomes in osteoarthritis: breakthrough innovations and advanced tissue engineering for cartilage regeneration since 2020. Biomedicines:2486. (2025) 13:2486. doi: 10.3390/biomedicines13102486. PMID: 41153769 PMC12562032

[60] Wu Y Li J Zeng Y Pu W Mu X Sun K . Exosomes rewire the cartilage microenvironment in osteoarthritis: from intercellular communication to therapeutic strategies. Int J Oral Sci. (2022) 14:40. doi: 10.1038/s41368-022-00187-z. PMID: 35927232 PMC9352673

[61] Zhang C Pan L Zhang H Ke T Yang Y Zhang L . Osteoblasts-derived exosomal lncRNA-MALAT1 promotes osteoclastogenesis by targeting the miR-124/NFATc1 signaling axis in bone marrow-derived macrophages. Int J Nanomedicine. (2023) 18:781–95. doi: 10.2147/ijn.s395607. PMID: 36814857 PMC9939803

[62] Pan B Zhang Z Wu X Xian G Hu X Gu M . Macrophage-derived exosomes modulate wear particle-induced osteolysis via miR-3470b targeting TAB3/NF-κB signaling. Bioact Mater. (2023) 26:181–93. doi: 10.1016/j.bioactmat.2023.02.028. PMID: 36911207 PMC9999169

[63] Liu J Wu X Lu J Huang G Dang L Zhang H . Exosomal transfer of osteoclast-derived miRNAs to chondrocytes contributes to osteoarthritis progression. Nat Aging. (2021) 1:368–84. doi: 10.1038/s43587-021-00050-6. PMID: 37117596

[64] Li B Ding T Chen H Li C Chen B Xu X . CircStrn3 targeting microRNA-9-5p is involved in the regulation of cartilage degeneration and subchondral bone remodeling in osteoarthritis. Bone Joint Res. (2023) 12:33–45. doi: 10.2139/ssrn.3935688. PMID: 36642417 PMC9872037

[65] Jin X Ding D Yan Y Li H Wang B Ma L . Phosphorylated RB promotes cancer immunity by inhibiting NF-κB activation and PD-L1 expression. Mol Cell. (2019) 73:22–35.e6. doi: 10.1016/j.molcel.2018.10.034. PMID: 30527665 PMC8968458

[66] Li X Wang Y Cai Z Zhou Q Li L Fu P . Exosomes from human umbilical cord mesenchymal stem cells inhibit ROS production and cell apoptosis in human articular chondrocytes via the miR-100-5p/NOX4 axis. Cell Biol Int. (2021) 45:2096–106. doi: 10.1002/cbin.11657. PMID: 34197004

[67] Wu X Crawford R Xiao Y Mao X Prasadam I . Osteoarthritic subchondral bone release exosomes that promote cartilage degeneration. Cells. (2021) 10:251. doi: 10.3390/cells10020251. PMID: 33525381 PMC7911822

[68] Mao G Xu Y Long D Sun H Li H Xin R . Exosome-transported circRNA_0001236 enhances chondrogenesis and suppress cartilage degradation via the miR-3677-3p/Sox9 axis. Stem Cell Res Ther. (2021) 12:389. doi: 10.1186/s13287-021-02431-5. PMID: 34256841 PMC8278601

[69] Li S Liu J Liu S Jiao W Wang X . Mesenchymal stem cell-derived extracellular vesicles prevent the development of osteoarthritis via the circHIPK3/miR-124-3p/MYH9 axis. J Nanobiotechnology. (2021) 19:194. doi: 10.1186/s12951-021-00940-2. PMID: 34193158 PMC8244143

[70] Xia Q Wang Q Lin F Wang J . miR-125a-5p-abundant exosomes derived from mesenchymal stem cells suppress chondrocyte degeneration via targeting E2F2 in traumatic osteoarthritis. Bioengineered. (2021) 12:11225–38. doi: 10.1080/21655979.2021.1995580. PMID: 34709978 PMC8809923

[71] Jin Y Xu M Zhu H Dong C Ji J Liu Y . Therapeutic effects of bone marrow mesenchymal stem cells-derived exosomes on osteoarthritis. J Cell Mol Med. (2021) 25:9281–94. doi: 10.1111/jcmm.16860. PMID: 34448527 PMC8500984

[72] Xu H Xu B . BMSC-derived exosomes ameliorate osteoarthritis by inhibiting pyroptosis of cartilage via delivering miR-326 targeting HDAC3 and STAT1//NF-κB p65 to chondrocytes. Mediators Inflammation. (2021) 2021:9972805. doi: 10.1155/2021/9972805. PMID: 34764819 PMC8577926

[73] Yan L Liu G Wu X . The umbilical cord mesenchymal stem cell-derived exosomal lncRNA H19 improves osteochondral activity through miR-29b-3p/FoxO3 axis. Clin Transl Med. (2021) 11:e255. doi: 10.1002/ctm2.255. PMID: 33463060 PMC7805401

[74] Kong R Gao J Zhang J Ji L Yu Y Zhang L . Synovial mesenchymal stem cell-derived exosomal miR-320c enhances chondrogenesis by targeting ADAM19. Future Med Chem. (2022) 14:81–96. doi: 10.4155/fmc-2021-0177. PMID: 34927445

[75] Lin Z Ma Y Zhu X Dai S Sun W Li W . Potential predictive and therapeutic applications of small extracellular vesicles-derived circPARD3B in osteoarthritis. Front Pharmacol. (2022) 13:968776. doi: 10.3389/fphar.2022.968776. PMID: 36339585 PMC9627215

[76] Kong R Zhang J Ji L Yu Y Gao J Zhao D . Synovial mesenchymal stem cell-derived exosomal microRNA-320c facilitates cartilage damage repair by targeting ADAM19-dependent Wnt signaling in osteoarthritis rats. Inflammopharmacology. (2023) 31:915–26. doi: 10.1007/s10787-023-01142-y. PMID: 36862227

[77] Li Q Zhao YH Xu C Liang YL Zhao Y He QM . Chemotherapy-induced senescence reprogramming promotes nasopharyngeal carcinoma metastasis by circRNA-mediated PKR activation. Adv Sci (Weinh). (2023) 10:e2205668. doi: 10.1002/advs.202205668. PMID: 36683218 PMC10015868

[78] Zou J Yang W Cui W Li C Ma C Ji X . Therapeutic potential and mechanisms of mesenchymal stem cell-derived exosomes as bioactive materials in tendon-bone healing. J Nanobiotechnology. (2023) 21:14. doi: 10.1186/s12951-023-01778-6. PMID: 36642728 PMC9841717

[79] Garcia-Martin R Wang G Brandão BB Zanotto TM Shah S Kumar Patel S . MicroRNA sequence codes for small extracellular vesicle release and cellular retention. Nature. (2022) 601:446–51. doi: 10.1038/s41586-021-04234-3. PMID: 34937935 PMC9035265

[80] Kijowski R Demehri S Roemer F Guermazi A . Osteoarthritis year in review 2019: imaging. (2020) 28:285–95. doi: 10.1016/j.joca.2019.11.009 31877380

[81] Chen A Chen Y Rong X You X Wu D Zhou X . The application of exosomes in the early diagnosis and treatment of osteoarthritis. Front Pharmacol. (2023) 14:1154135. doi: 10.3389/fphar.2023.1154135. PMID: 37188263 PMC10175594

[82] Wang C Li N Liu Q Su L Wang S Chen Y . The role of circRNA derived from RUNX2 in the serum of osteoarthritis and its clinical value. J Clin Lab Anal. (2021) 35:e23858. doi: 10.1002/jcla.23858. PMID: 34165827 PMC8274987

[83] Zhao Y Xu J . Synovial fluid-derived exosomal lncRNA PCGEM1 as biomarker for the different stages of osteoarthritis. Int Orthop. (2018) 42:2865–72. doi: 10.1007/s00264-018-4093-6. PMID: 30128669

[84] Dong X Niu Y Ding Y Wang Y Zhao J Leng W . Formulation and drug loading features of nano-erythrocytes. Nanoscale Res Lett. (2017) 12:202. doi: 10.1186/s11671-017-1980-5. PMID: 28314369 PMC5355415

[85] Luo P Jiang C Ji P Wang M Xu J . Exosomes of stem cells from human exfoliated deciduous teeth as an anti-inflammatory agent in temporomandibular joint chondrocytes via miR-100-5p/mTOR. Stem Cell Res Ther. (2019) 10:216. doi: 10.1186/s13287-019-1341-7. PMID: 31358056 PMC6664713

[86] Cosenza S Ruiz M Toupet K Jorgensen C Noël D . Mesenchymal stem cells derived exosomes and microparticles protect cartilage and bone from degradation in osteoarthritis. Sci Rep. (2017) 7:16214. doi: 10.1038/s41598-017-15376-8. PMID: 29176667 PMC5701135

[87] Rizzi L Turati M Bresciani E Anghilieri FM Meanti R Molteni L . Characterization of microRNA levels in synovial fluid from knee osteoarthritis and anterior cruciate ligament tears. Biomedicines. (2022) 10:2909. doi: 10.3390/biomedicines10112909. PMID: 36428476 PMC9687202

[88] Piffoux M Volatron J Cherukula K Aubertin K Wilhelm C Silva AKA . Engineering and loading therapeutic extracellular vesicles for clinical translation: a data reporting frame for comparability. Adv Drug Delivery Rev. (2021) 178:113972. doi: 10.1016/j.addr.2021.113972. PMID: 34509573

[89] Liang Y Xu X Li X Xiong J Li B Duan L . Chondrocyte-targeted microRNA delivery by engineered exosomes toward a cell-free osteoarthritis therapy. ACS Appl Mater Interfaces. (2020) 12:36938–47. doi: 10.1021/acsami.0c10458. PMID: 32814390

[90] Zhao S Xiu G Wang J Wen Y Lu J Wu B . Engineering exosomes derived from subcutaneous fat MSCs specially promote cartilage repair as miR-199a-3p delivery vehicles in osteoarthritis. J Nanobiotechnology. (2023) 21:341. doi: 10.1186/s12951-023-02086-9. PMID: 37736726 PMC10515007

[91] Tao SC Huang JY Gao Y Li ZX Wei ZY Dawes H . Small extracellular vesicles in combination with sleep-related circRNA3503: a targeted therapeutic agent with injectable thermosensitive hydrogel to prevent osteoarthritis. Bioact Mater. (2021) 6:4455–69. doi: 10.1016/j.bioactmat.2021.04.031. PMID: 34027234 PMC8120802

[92] Foo JB Looi QH How CW Lee SH Al-Masawa ME Chong PP . Mesenchymal stem cell-derived exosomes and microRNAs in cartilage regeneration: biogenesis, efficacy, miRNA enrichment and delivery. Pharm (Basel). (2021) 14:1093. doi: 10.3390/ph14111093. PMID: 34832875 PMC8618513

