# Correction: Roles of exosomal non-coding RNAs in osteoarthritis

**DOI:** 10.3389/fimmu.2026.1881307

**Published:** 2026-05-26

**Authors:** Jing Lu, Jiayou Wen, Jing Ji

**Affiliations:** 1 School of Traditional Chinese and Western Medicine, Gansu University of Chinese Medicine, Lanzhou, Gansu, China; 2 Department of Rehabilitation Medicine, Gansu Provincial Hospital of Traditional Chinese Medicine, Lanzhou, Gansu, China

**Keywords:** biomarkers, cartilage degradation, exosomes, inflammation, non-coding RNAs, osteoarthritis

Affiliation “School of Traditional Chinese and Western Medicine, Gansu University of Chinese Medicine, Lanzhou, Gansu, China” was omitted for author “Jiayou Wen”. This affiliation has now been added for author “Jiayou Wen”.

Author “Jiayou Wen” was erroneously assigned to affiliation “Department of Rehabilitation Medicine, Gansu Provincial Hospital of Traditional Chinese Medicine, Lanzhou, Gansu, China”. This affiliation has now been removed for author “Jiayou Wen”.

The original version of this article has been updated.

